# A review: Pancreatic enzymes in the treatment of chronic pancreatic insufficiency in companion animals

**DOI:** 10.1111/jvim.17096

**Published:** 2024-05-09

**Authors:** Dominika Szkopek, Stefan G. Pierzynowski, Kateryna Pierzynowska, Kamil Zaworski, Agata Kondej, Piotr Wychowański, Paweł Konieczka, Blanka Seklecka, Janine Donaldson, Michał Jank, Jarosław Woliński

**Affiliations:** ^1^ Laboratory of Large Animal Models, The Kielanowski Institute of Animal Physiology and Nutrition Polish Academy of Sciences, Instytucka 3 Jabłonna Poland; ^2^ Anara AB Trelleborg Sweden; ^3^ Department of Medical Biology Witold Chodźka Institute of Rural Medicine Lublin Poland; ^4^ Department of Biology Lund University Lund Sweden; ^5^ Department of Animal Physiology, The Kielanowski Institute of Animal Physiology and Nutrition Polish Academy of Sciences, Instytucka 3 Jabłonna Poland; ^6^ Oral Surgery and Implantology Unit, Division of Oral Surgery and Implantology, Department of Head and Neck, Institute of Clinical Dentistry Fondazione Policlinico Universitario A. Gemelli IRCCS, Universita Cattolica del Sacro Coure Rome Italy; ^7^ Department of Animal Nutrition, The Kielanowski Institute of Animal Physiology and Nutrition Polish Academy of Sciences, Instytucka 3 Jabłonna Poland; ^8^ Department of Poultry Science and Apiculture University of Warmia and Mazury, Oczapowskiego 5 Olsztyn Poland; ^9^ Phase Research Team, Adamed Pharma S.A. Pieńków Poland; ^10^ School of Physiology, Faculty of Health Sciences University of the Witwatersrand Parktown, Johannesburg 2193 South Africa; ^11^ Department of Pre‐Clinical Sciences and Infectious Diseases, Faculty of Veterinary Medicine and Animal Science Poznan University of Life Sciences Poznań Poland

**Keywords:** animal‐derived enzymes, exocrine pancreatic insufficiency, microbial‐derived enzymes, pancreatic replacement therapy

## Abstract

The purpose of this review was to analyze the scientific literature on exocrine pancreatic insufficiency (EPI) in dogs and cats and our own research on porcine model to compare animal‐ and microbial‐derived enzymes in the treatment of animals with this disease. Clinical signs of EPI occur when more than 85% of the pancreatic parenchyma is non‐functional. EPI can be a consequence of various diseases. The insufficient activity or deficiency of pancreatic enzymes leads to impaired digestion and absorption, and consequently, to malnutrition. The primary treatment for enzyme insufficiency is pancreatic enzyme replacement therapy (PERT). PERT in animals with EPI is a lifetime therapy. Most commercially available products are of animal origin (processed pancreata obtained from a slaughter house) and contain lipases, alpha‐amylase, and proteases. Enzymes of microbial and plant origin seem to be a promising alternative to animal‐derived enzymes, but to date there are no registered preparations containing all enzymes simultaneously for use in clinical practice to treat EPI. Results from some previous studies have highlighted the “extra‐digestive” functions of pancreatic enzymes, as well as the actions of pancreatic‐like microbial enzymes. For example, trypsin activates protease‐activated receptor and provokes maturation of enterocytes and enterostatin inhibits fat absorption. It has been postulated that intrapancreatic amylase is the main component of the acini‐islet‐acinar axis—the reflex which down regulates insulin release, while gut and blood amylase exhibit anti‐incretin actions “per se.” Additionally, high but still physiological blood amylase activity coincide with physiological glucose homeostasis and a lack of obesity.

**Abbreviations**
EPI: exocrine pancreatic insufficiency
PERT: pancreatic enzyme replacement therapy
PLEM: pancreatic enzymes of microbial origin

## 1 INTRODUCTION

Insufficiency of the exocrine pancreas can be a consequence of various diseases, such as atrophy of pancreatic acinar cells, chronic pancreatitis, pancreatic cancer, cystic fibrosis, severe acute necrotizing pancreatitis or gastrointestinal and pancreatic surgical resections.^1^ They all share a common pathophysiological result: insufficient activity or deficiency of pancreatic enzymes, which causes digestive and absorption disorders. The primary treatment for enzymatic insufficiency is pancreatic enzyme replacement therapy (PERT). This is lifelong therapy. Both, human and veterinary products contain enzymes of animal origin (pancreatin from porcine pancreas). In recent years, there has been increasing interest in the use of enzymes of microbial origin, for many reasons, including production and their action. Enzymes of microbial and plant origin appear to be a promising alternative to animal‐derived enzymes, but to date there are no registered preparations containing all enzymes simultaneously for use in clinical practice.^2^, ^3^, ^4^, ^5^, ^6^ In addition, interestingly, some pancreatic enzymes, also microbial‐derived, have so‐called hormonal functions, for example, amylase,^7^ which can be used for the treatment of obesity.

A number of studies on the effects of microbial‐derived enzymes show similar or even better effects compared to animal‐derived enzymes, prompting thoughts on expanding research on this topic.^8^, ^9^, ^10^, ^11^, ^12^, ^13^, ^14^, ^15^ Also, it seems appropriate to focus on the “extra‐digestive” functions of pancreatic‐like microbial enzymes.

## 2 PATHOPHYSIOLOGY OF THE EXOCRINE PANCREAS

A healthy pancreas effectively protects itself from self‐digestion by a number of mechanisms, including the synthesis, storage and secretion of enzymes as inactive zymogens (proenzymes), which are normally activated when secreted into the lumen of the small intestine. It also produces trypsin enzyme inhibitors (PTSI), which protect it from minimal, albeit harmful (“accidentally” activated) amounts of enzymes inside it. When protective mechanisms are damaged or blocked, pancreatitis can occur. It can have an acute (mild or severe and life‐threatening) or chronic course that can occur in a subclinical form until about 90% of the pancreatic tissue is destroyed and exocrine pancreatic insufficiency (EPI) develops, because of insufficient secretion of digestive enzymes and the related impairment of digestion and, consequently, the absorption of macronutrients.^16^

In adult animals and humans, the functional reserve of the pancreas is considerable, and clinical signs of EPI appear when more than 85% of the pancreatic parenchyma is inefficient.^17^ In growing mammals—especially during the juvenile period—maximum enzymatic secretion is needed to maintain proper growth.^18^, ^19^

There are alternative pathways for the digestion of certain nutrients. After experimentally cutting off the supply of pancreatic juice, dogs can still absorb up to 63% of the protein and 84% of the fat they ingest.^16^ This activity is likely derived from gastric lipases and pepsins, esterases and peptidases of the intestinal mucosa, and in young animals from milk bile salt activated lipase. However, if exocrine pancreatic function is relevant impaired, these alternative routes prove insufficient, and signs of malabsorption appear. EPI can be congenital or acquired. Among the causes of disorders of the exocrine pancreas, they can be distinguished as atrophy of pancreatic acinar cells (PAA), pancreatic nodular hyperplasia, tumors, hypoplasia, aplasia, congenital abnormalities, pancreatitis, and pancreatic necrosis.

The most well‐documented breed predisposition is the German Shepherds (GSDs), and while any breed can be affected, Collies, Cavalier Kings Charles Spaniels, Chow Chows, West Highland White Terriers, Eurasier dogs and Cairn Terriers are reported to be at increased risk of EPI.^20^, ^21^, ^22^, ^23^, ^24^

EPI can be developed in dogs of almost any age, with a reported age range of 3 months to 17 years,^1^ but the underlying etiology can affect the age of onset of clinical signs.^25^ Considering all dogs with EPI, the disease is more likely to occur in young adult dogs (median age 3 years).^1^, ^16^ Females and obese animals are more susceptible to EPI (female dogs account for about 56.5% of dogs).^1^

EPI in cats is less common than in dogs, but increasing fTLI (feline trypsin‐like immunoreactivity) studies have shown that the disease could be much more common than previously thought.^16^, ^26^ EPI can appear in cats of any age, with some diagnosed at less than 1 year old. However, EPI is more commonly diagnosed in middle‐aged and older cats, with an estimated average age of 8 years.^27^, ^28^ No sex preference has been found in cats,^29^ but according to some studies males are slightly overrepresented in cases of EPI.^27^, ^28^

## 3 PANCREATIC ENZYMES AND THEIR ACTIVITY

### 3.1 Lipases

This is a group of hydrolase enzymes. In addition to pancreatic lipase, which is the most important of the lipases, there are also gastric, lipoprotein, intestinal, and hormone‐sensitive lipases. Pancreatic lipase (triacylglycerol acyl hydrolase) is an enzyme that plays a key role in the process of dietary fat absorption. It is responsible for the hydrolysis of triglycerides into di‐ and monoglycerides and fatty acids. Bile salts, which allow the enzyme dissolved in water to come into contact with fats, and colipase, which supports the lipolytic properties of lipase and adapts it to the acidic environment in which it is inactivated, are important in its functioning.^30^ Only pancreatic lipases can cleave polyunsaturated fatty acids (PUFA) which in turn are indispensable among others for brain and vision development. It is also important to point out that erythrocyte contents of PUFA which is related to dietary fat can be important diagnostic tools for estimation of animal health.^31^ Pancreatic lipases need the presence of active pancreatic procolipase for their conjugation with bile salts.^32^ The ratio of lipase and procolipase secreted by the pancreas is species specific. It is postulated that the activated pentapeptide of procolipase, enterostatin, inhibits fat absorption, and regulates pancreatic juice secretion.^33^

### 3.2 Amylases

Also known as diastases, amylolytic enzymes. Amylases are hydrolases, including both pancreatic and salivary amylase (formerly called ptyalin). Their function is to break down starch and other polysaccharides. The pancreas and salivary glands have amylase concentrations several orders of magnitude higher than those in any other tissue and probably account for virtually all serum amylase activity in healthy individuals.^34^ Interestingly, amylases are also synthesized in the fruits of many plants during their ripening and germination of cereal grains. There are 3 types of amylases—α, β, and γ. Types α and γ have been found in animals and humans. The mechanism of action of α‐amylase is the hydrolysis of α‐(1,4)‐glycosidic bonds of amylose to maltose molecules. Recent studies from our lab pointed out various important, parallel, non‐enzymatic functions of amylase. The first 1 is the intrapancreatic integrative action of amylase on insulin release/secretion,^7^, ^35^ where amylase, directly from acinar cells, inhibits insulin secretion. The second non‐enzymatic action of amylase is its anti‐incretin action within the duodenum—the feature which forms the background of the “miracle” bariatric surgery, which eliminates diabetes type 2.^7^, ^35^ Thus, it is not surprising that low blood levels of amylase in humans is coincidental with obesity.^36^

### 3.3 Proteases

Also known as peptidases, proteolytic enzymes. Proteases are enzymes from the class of hydrolases which catalyze proteolysis. Their functions include the hydrolysis of peptide bonds. Peptidases can be divided into 2 groups—endopeptidases, which cleave peptide bonds within the peptide chain, and exopeptidases, which cleave single amino acids from the ends of the chain. Due to the catalysis mechanism, the following are distinguished: serine and cysteine proteases. Protease inhibitors are used as therapeutic agents in medicine. These are natural or synthetic substances which inhibit the activity of proteolytic enzymes. There are specific and non‐specific inhibitors. They can be protein or non‐protein. Pancreatic endopeptidases are involved in the so‐called plastein reaction in the intestinal contents, involving the attachment of amino acids to peptides and whole proteins (transformation of proteins into polypeptides with a mass of about 3 kDa), which very often results in reduction of allergic potential of peptides or proteins.^37^

Trypsin, parallel to enzymatic digestive and autocatalytic features, exhibits an extraordinary regulatory capacity. In the early 70s, Rothman recognized trypsin as a hormone.^38^ Previous studies by our lab have shown that proteases stimulate the precocious maturation of enterocytes.^39^ Nowadays, trypsin is recognized as a potent PAR receptor inactivator,^40^ actively participating in the inflammatory response.

## 4 PANCREATIC ENZYME REPLACEMENT THERAPY

### 4.1 General information about PERT in dogs and cats

Insufficiency of the exocrine pancreas can be a consequence of various diseases which all share a common pathophysiological result: insufficient activity or deficiency of pancreatic enzymes, which causes digestive and absorption disorders. The basis of treatment for enzyme failure is PERT. Approximately 60% to 65% of dogs respond well to enzyme therapy, partial in 17% and poor in 23%^41^, ^42^, ^43^ and 40% to 60% of cats have complete and 27% to 60% have partial responses to therapy.^25^, ^27^, ^44^

Regardless of the product used, enzymes are added to every meal. Pre‐incubation of food with enzymes or supplementation with bile salts is not necessary, regardless of the source of the enzymes.^45^ The starting dose varies depending on the manufacturer, animal's weight and dietary fat content, but is usually adjusted individually after initial treatment and resolution of clinical signs. Once signs subside, the dose of pancreatic enzymes given with each meal can be gradually reduced to the lowest effective dose. PERT in animals with EPI is required for life.

### 4.2 Animal‐derived enzymes

Enteric‐coated preparations, uncoated enzymes in powder or capsule form, and raw pancreas supplements are available. Table 1 shows the options for using animal‐derived enzymes to treat dogs and cats with EPI. Most products contain pancreatic enzymes and proenzymes derived from pork pancreas in the form of dried extracts. The first pancreatic enzyme supplements came in the form of powder and tablets. However, they were only partially effective in alleviating EPI signs in people.^46^ Pancreatic enzymes operate in the alkaline environment of the duodenum. Up to 85% of pancreatic enzyme activity can be lost in the stomach because of the low pH or action of gastric pepsins.^47^ Orally administered preparations, if not coated, are broken down not only by acid and pepsin, but also, after being released into the duodenum, by the enzymes themselves, especially lipase.^48^ Therefore, changes were introduced to the existing recipes of preparations and coated enteric microspheres, as well as modifications of enzymes to protect them against inactivation in the stomach, which increased their effectiveness. Dogs receiving an enteric‐coated product containing pancreatic enzymes responded better to treatment compared to dogs receiving an identical but uncoated product.^49^ Other studies suggest no difference in efficacy between coated and noncoated preparations in dogs,^41^, ^50^ but enteric‐coated enzymes are considered in refractory cases when increasic doses of non‐enteric‐coated enzymes are ineffective.^25^ Moreover, microsphere preparations are characterized by increased mixing with the food content compared to tablets.^48^ The optimal particle size is ≥2 mm in diameter.^51^

**TABLE 1:** Pancreatic enzymes options for dogs and cats with exocrine pancreatic insufficiency.

	Dogs	Cats	Notes
Pancreatic enzymes			
Powder	1‐2 teaspoons per 10 kg of body weight in each meal	0.5‐1 teaspoon per meal	Mix with food just before feeding
Coated preparations for enteral administration	Coated preparations for enteral administration. The amount of coated preparations should be gradually reduced until the minimum effective dose is established when clinical signs are in remission		Administer with food
Raw pancreas	50‐100 g per meal, mixed with food	30‐90 g per meal, mixed with food	Rarely it can cause bleeding and irritation of the oral cavity, in such cases it is advisable to reduce the dose

Currently, human, and veterinary preparations are used in animals suffering from EPI. They contain the so‐called pancreatin, an extract from pork pancreas, which includes lipase, amylase, and proteases. Pancreatin was used in medicine already in the 19th century, and because of its effectiveness and safety, it is on the WHO (World Health Organization) list of drugs. The preparations differ in their quantitative composition, that is, the content of individual enzymes, and their qualitative composition. The activity of enzymes in preparations, depending on the part of the world, can be expressed in Ph. Eur.—unit of enzymatic activity according to the European Pharmacopoeia or USP—international unit of enzymatic activity.

For cats that sometimes cannot tolerate the taste of pancreatic powder, the enzymes should be thoroughly mixed with the food or fish oil added. It is optimal to take half the dose at the beginning of a meal and the rest during the meal. This greatly improved mixing of enzymes with food content in the stomach.^52^ Please note that crushing, chewing, or holding pancreatin capsules in the mouth could cause local irritation. In human medicine, extracts without a capsule should only be administered to patients after gastrectomy.^53^

Raw pancreas is usually obtained from cattle or pigs. It contains pancreatic enzymes and can be used to treat animals with EPI. It should be frozen in portions, with each portion used for 1 meal. This is an economical method of pancreatic enzyme supplementation, but it has some limitations. First, processing raw pancreas can be difficult and burdensome for owners, and second—and more importantly—raw animal tissues can be contaminated with pathogens, for example *Salmonella* spp. or *Campylobacter* spp., and thus pose a risk of infection for both animals and people. For these reasons, this method is not commonly used.

In the United States, bovine pancreatic enzymes are available in the form of an oral powder for dogs and cats (eg, Canine Ceuticals, Organic Beef Pancreas or Thrive, Beef Pancreas). According to Canine Ceuticals, a teaspoon of freeze‐dried pancreas is equivalent to approximately 10 g of raw pancreas. The Thrive company provides the specific activity of individual enzymes in the composition: 700 USP units of lipase, 250 000 USP units of amylase, and 25 000 USP units of protease. The product contains freeze‐dried bovine pancreas. Powder production is based on freeze‐drying. The process involves removing water from a frozen product by ice sublimation, that is, the ice turns directly into water vapor, bypassing the liquid state. Lyophilisates are obtained in the form of amorphous powders with a large surface area, which means that they dissolve faster than crystalline preparations.

Compared to porcine enzymes, purified bovine lipase has partial activity against emulsified short‐ and long‐chain triglycerides.^54^ Maximum activity is achieved only after adding a fraction with properties analogous to colipase.^54^ In chicken pancreas lipase and amylase activities decreased with increasing proteolytic activity.^55^

Regulations and safety considerations, in addition to microbiological purity, require the removal or inactivation of viruses in biopharmaceuticals such as pancreatin. Evaluation of the production process for the inactivation and/or removal of contamination with viruses, especially zoonotic ones, such as, for example, hepatitis E virus, pseudorabies virus (PRV), parvovirus (PPV), encephalomyocarditis virus (EMCV), bovine viral diarrhea virus (BVDV) or norovirus, is necessary to ensure the viral safety of biopharmaceuticals of animal origin.^56^

### 4.3 Microbial‐ and plant derived enzymes

Enzymes of microbial and plant origin seem to be a promising alternative to enzymes of animal origin.^2^, ^3^, ^4^, ^5^, ^6^, ^8^, ^9^, ^10^, ^11^, ^12^, ^13^, ^14^, ^15^ Currently, however, there are no registered preparations containing all enzymes simultaneously for use in clinical practice. Only about 2% of microorganisms have been tested as enzyme sources. Many plant lipases have been isolated and can be used to produce important lipases.^57^ Synergistic effects have been observed using a combination of animal‐derived and microbial‐derived enzymes.^3^

Microbial lipases constitute an important group of biotechnologically valuable enzymes, mainly because of the versatility of their functional properties and ease of mass production. Among others, Sigma‐Aldrich, an American biotechnology company, offers lipases, amylases, and proteases of microbial and plant origin in the form of powder, solid or physiological saline solution (biuret). Examples of lipases are wheat germ lipase (powder, 5‐15 U/mg; U—enzyme unit), *Aspergillus niger* lipase (powder, 200 U/g), *Candida rugosa* lipase (powder, >40 000 U/mg proteins), *Rhizopus oryzae* lipase (powder, 10 and >30 U/mg), and *Pseudomonas cepacia* lipase (powder, >30 U/mg). Amylases include *Aspergillus oryzae* α‐amylase (biuret, >150 U/mg protein, powder, 30 U/mg), *Bacillus licheniformis* α‐amylase (powder, 500‐1500 units/mg protein, saline solution, >500 U/mg protein), barley β‐amylase (biuret, 20‐80 units/mg protein). Proteases include *Aspergillus saitoi* protease (>0.6 U/mg solid), *Streptomyces* protease (>15 U/mg solid), and *Aspergillus oryzae* protease (>500 U/g).

Lipase is of particular interest in scientific research. Because most of the nutritional problems in EPI are secondary to the improper digestion of dietary fats, many researchers have focused on the identification of recombinant microbial lipases.^6^ Microbial lipases are highly diverse in terms of their enzymatic properties and substrate specificity, which makes them attractive to many industrial sectors, such as cosmetics, chemicals, food, textiles, and pharmaceuticals. It is believed that because of, among others, the ease of mass production (availability of large amounts of purified lipase), ease of genetic manipulation, greater stability, activity in ambient conditions, which eliminates the energy expenditure required to carry out the reaction at elevated temperature and pressure, reduction of post‐reaction separation problems, more convenient and safer production and activity in organic solvents can be more useful than enzymes of plant or animal origin. Bacterial strains are used more often because they have higher activity compared to fungi and usually have an optimal pH—neutral or alkaline and are often thermostable.^57^

In the pharmaceutical industry, lipases are used as modulators, such as activators and inhibitors, especially to treat lifestyle diseases, such as obesity. Currently, modulators are of great importance in therapies and will surely be further developed soon.^58^ A general disadvantage of industrial lipase production is its cost because of expensive carbon and nitrogen sources, which account for approximately 50% of the total enzyme production costs.^59^ A study assessing the effect of bacterial lipase and porcine lipase on the absorption of carbohydrates, fats and proteins in dogs with EPI^13^ shows that eliminating steatorrhea required 75 times more porcine lipase than bacterial lipase (18 g vs 240 mg) and that high‐fat and high‐protein diets optimize fat absorption using both enzymes.

Exogenous microbial enzymes mimic endogenous pancreatic enzymes recovered in the lumen of the gastrointestinal tract which was confirmed in a pig model with induced EPI.^15^ Another study was based on testing a new approach to the treatment of EPI with a mixture of pancreatic enzymes (lipase, amylase, and protease in the form of powder from Sigma‐Aldrich) of microbial origin (PLEM) in pigs with EPI. In this study, in addition to the coefficient of fat and nitrogen absorption (CFA, CNA), the lipemic index (LI), plasma triglyceride (TG) and non‐esterified fatty acid (NEFA) concentrations, and somatic growth were also examined. The results showed that PLEM improved the digestion of fat and protein, CFA and CNA increased by 59% and 43%, respectively, and the postprandial blood lipid profile was normal as in healthy animals, although the absorption of fat and protein was lower than in the control group. This leads to the conclusion that PLEM supports somatic growth and normalizes the postprandial lipid profile.^60^

Novel microbial lipase (NM‐BL) contains only lipase, which might limit its use as a sole PERT in animals and humans with pancreatic insufficiency.^6^ It is produced through fermentation using *Burkholderia plantarii*, which is not pathogenic to humans. Unlike PERT available on the market, NM‐BL is a liquid preparation, so it can be more convenient to administer than conventional PERT. In vitro studies have shown that NM‐BL is more resistant to inactivation by gastric acid and proteases than pancreatin‐containing products, and in dogs with experimentally induced pancreatic insufficiency, the effectiveness in reducing steatorrhea was comparable to that of pork products.^13^ To further investigate NM‐BL for pancreatic enzyme replacement, a controlled phase IIa clinical trial was conducted to determine its safety and efficacy in dogs and cats with cystic fibrosis and EPI. Based on the literature no further information about NM‐BL is available.

Another example is adrulipase. It is a recombinant lipase produced by the yeast species *Yarrowia lipolytica*, manufactured in a biotechnological process. To evaluate its effectiveness compared to existing pancreatic enzyme supplements, studies will be performed, such as coefficient of fat absorption (CFA), fecal weight, signs of malabsorption, and coefficient of nitrogen absorption (CNA).

A novel approach is the attempt to use virus‐like nanoparticles (VLP) as enzyme carriers to improve enzyme replacement therapy.^61^ Virus‐like nanoparticles can display highly concentrated enzymatic activity. The porous structure enables the entry of metabolites and the exit of products. Moreover, the presence of specific ligands on the surface facilitates functionalization through recognition by specific cellular receptors to avoid systemic circulation. Additionally, their surface can be covered with non‐immunogenic proteins or polymers, making the nanoparticles invisible to the immune system.^62^ These features make VLPs a potential candidate for metabolic activation of prodrugs to pharmacologically active forms. This is the so‐called enzyme prodrug therapy (EPT). Thanks to this, they can be specifically delivered to cancer cells, increasing drug activation on site, reducing side effects and increasing the effectiveness of treatment.^61^

### 4.4 Hormonal functions of pancreatic enzymes

It should be mentioned that some pancreatic enzymes, apart from their enzymatic function, also have so‐called hormonal functions. Administration of exogenous protease‐containing enzymes induces premature intestinal maturation in suckling rats, confirming that proteases exert effects on intestinal mucosal maturation.^39^ Another example is the anti‐incretin effect of enterally administered amylase. Incretins are protein hormones responsible for postprandial insulin release. Amylase can be responsible for reducing the absorption of glucose into the blood and, consequently, reducing the release of insulin.^35^ The results of studies conducted on pigs indicate that the intestines, stimulated by amylase or its peptides, actively participate in the regulation of the amount of glucose absorbed into the blood and that which is metabolized by the intestinal tissues. Reducing glucose absorption into the blood while limiting insulin release provides evidence for an intestinal amylase‐dependent mechanism that regulates blood glucose concentrations in an insulin‐independent manner. This ability of amylases can help prevent the development of obesity and diabetes.^35^, ^63^

## 5 CONCLUSIONS

The pancreas is a glandular organ with both endocrine and exocrine functions. The exocrine pancreas produces enzymes, including lipase, amylase, and protease, which are part of the pancreatic juice and are involved in the digestion of fats, carbohydrates, and proteins, respectively function disorders with its functioning can negatively affect the health status, in both humans and animals. Signs of EPI occur when more than 85% of the pancreatic parenchyma is non‐functional. EPI can be a consequence of various diseases, all of which share a common pathophysiological result, which is insufficient activity or deficiency of pancreatic enzymes that leads to impaired digestion and absorption. The primary treatment for enzyme insufficiency is PERT. PERT in animals with EPI is a lifetime therapy. The main factor influencing the effectiveness of PERT therapy is compliance. In animals that respond positively to the treatment, the prognosis is good. Most commercially available products are of animal origin and contain pancreatin, an extract from pig pancreases containing lipase, amylase, and protease. Enzymes of microbial and plant origin seem to be a promising alternative to animal‐derived enzymes, but to date there are no registered preparations containing all enzymes simultaneously for use in clinical practice. A number of studies on the effects of enzymes of microbial origin show beneficial effects in animals and humans with EPI, similar or even better compared to enzymes of animal origin. Currently, only about 2% of microorganisms have been tested as enzyme sources. Due to, among other things, ease of mass production and genetic manipulation, greater stability, activity under ambient conditions (elimination of energy expenditure to carry out the reaction at elevated temperature and pressure), reduced post‐reaction separation problems, more convenient and safer production, and activity in organic solvents, they can be more useful than enzymes of plant or animal origin. In addition, interestingly, some pancreatic enzymes also have so‐called hormonal functions, for example, amylase or trypsin. It is important that high but still physiological blood amylase levels coincide with physiological glucose homeostasis and a lack of obesity. Based on this, it can be thought that future research should focus on microbial‐derived enzymes for the treatment of EPI or other diseases, for example obesity or diabetes, and the development of formulations containing all the necessary enzymes for the pharmaceutical industry which will help to improve current therapies in companion animals.

## CONFLICT OF INTEREST DECLARATION

Authors declare no conflict of interest.

## OFF‐LABEL ANTIMICROBIAL DECLARATION

Authors declare no off‐label use of antimicrobials.

## INSTITUTIONAL ANIMAL CARE AND USE COMMITTEE (IACUC) OR OTHER APPROVAL DECLARATION

Authors declare no IACUC or other approval was needed.

## HUMAN ETHICS APPROVAL DECLARATION

Authors declare human ethics approval was not needed for this study.
